# Quality of Life and Metabolic Indicators of Patients with Type 2 Diabetes: A Cross-Sectional Study in Iran

**DOI:** 10.1155/2022/4046012

**Published:** 2022-12-28

**Authors:** Mohammad Hossein Kaveh, Keramat Noori, Mahin Nazari, Khadijeh Khademi

**Affiliations:** ^1^Research Center for Health Sciences, Institute of Health, Department of Health Promotion, School of Health, Shiraz University of Medical Sciences, Shiraz, Iran; ^2^Department of Health Promotion, School of Health, Shiraz University of Medical Sciences, Shiraz, Iran; ^3^Student Research Committee, Department of Health Promotion, School of Health, Shiraz University of Medical Sciences, Shiraz, Iran

## Abstract

**Background:**

The World Health Organization (WHO) has considered type 2 diabetes mellitus (T2DM) a major global health challenge because of its high prevalence worldwide. T2DM can affect patients' personal, social, and economic statuses. On the other hand, due to the increasing prevalence of T2DM, Quality of Life (QOL) has received more attention in recent years.

**Objective:**

The present study was conducted to investigate the relationships between QOL and physical activity level, body mass index, fasting blood sugar, high-density lipoprotein cholesterol, low-density lipoprotein cholesterol, triglyceride, total cholesterol, HbA1c, and systolic and diastolic blood pressure among Iranian patients with uncomplicated T2DM.

**Methods:**

This cross-sectional study was conducted on 135 participants selected through consecutive sampling. The study data were collected using International Physical Activity Questionnaire and Short-Form Health Survey Questionnaire. Then, the data were entered into the SPSS ver. 28 software, and Pearson's correlation was used to measure the correlation between the variables. Linear regression was also employed. The significance level was set at 0.05.

**Results:**

The significant association was observed between gender (*p* = 0.007), HDL level (*p* = 0.02), and gender-adjusted physical activity (*p* = 0.002) with QOL.

**Conclusions:**

Due to the association between physical activity and HDL level with QOL in patients with uncomplicated T2DM, they should be given the necessary training to improve their physical activity and regulate HDL level. Also, empowering them in this matter improves their QOL.

## 1. Introduction

The World Health Organization (WHO) has considered type 2 diabetes mellitus (T2DM) a major global health challenge due to its high prevalence worldwide [1]. There are currently more than 423 million patients [2], the majority of whom live in developing countries [3]. Based on the World Health Report, the prevalence of T2DM in adults has been expected to increase by 64% between 1995 and 2045 (from 135 million to 628 million) [4]. Accordingly, there will be a 42% increase from 51 to 72 million in developed countries and a 170% increase from 84 to 228 million in developing countries. Thus, the proportional increase will be greater in developing countries [2, 5, 6]. In Iran, the prevalence of T2DM is approximately 11.9%. It has also been estimated that 9.2 million patients will suffer from T2DM by 2030 [7].

Studies performed in Iran and some other countries have indicated that T2DM negatively influences the patients' Quality of Life (QOL) [8–12]. T2DM is, in fact, a costly health problem for patients and the healthcare system that is detected in all age groups and countries [2]. It can affect the personal, social, and economic statuses of patients, increase the prevalence of chronic diseases and dissatisfaction with life, and decrease the QOL. In addition, boring treatments as well as disabling and life-threatening complications of T2DM can affect patients' physical, psychological, and social health, i.e., QOL [13–16]. Since patients' QOL may boost their metabolism [17], more attention should be paid to the key determinants of Health-Related QOL (HRQOL) to identify and implement appropriate policies to achieve better T2DM management and improve the patients' QOL [8]. In this context, it is necessary to determine how T2DM affects patients' QOL since improving the QOL is among the most important goals of healthcare systems [18].

Studies have indicated that T2DM negatively influences the QOL, but the level of influence has been different across various studies [8–16]. Additionally, contradictory results have been obtained regarding the relationship among QOL, physical activity, and some metabolic indicators, such as body mass index (BMI), triglyceride, hemoglobin A1c (HbA1c), and high-density lipoprotein (HDL) cholesterol that seem to be associated with T2DM [19–22].

Thus, the present study aims to investigate the relationships between QOL and physical activity level, BMI, FBS, HDL, LDL, triglyceride, total cholesterol, HbA1c, and systolic and diastolic blood pressure among Iranian patients with T2DM.

## 2. Methods

### 2.1. Study Design, Setting, and Period

This cross-sectional, comparative study was conducted on the participants selected through consecutive sampling of patients with T2DM who had active medical records in Mobarakeh Diabetes Clinic affiliated with the Social Security Organization during January–March 2019.

### 2.2. Sample Size Determination and Study Participants

The sample size was estimated at a confidence interval of 95% (*z*_1−(∝/2)_=1.96) with an acceptable error (*d*) of 1.85 (3% mean: 61.90) and a standard deviation (*σ*) of 7.50 for the Quality of Life of Iranian patients with T2DM [23, 24]. Then, 135 were included in the study due to the probability of not completing the questionnaires. The inclusion criteria were (1) no mental retardation and (2) writing and reading literacy, and the exclusion criteria included suffering from (1) other diseases or disorders such as cancer, multiple sclerosis, arthritis, dementia, and depression and (2) complications such as diabetes-related neuropathy and nephropathy.

### 2.3. Data Collection

The data including metabolic indices, FBS, HDL, LDL, triglyceride, total cholesterol, and HbA1c were extracted from the patients' medical records within 30 days before the survey. Other data including weight and height for estimated BMI and blood pressure were recorded by a specialist on the day the patients attended the clinic for care and treatment. Then, the questionnaires were completed in a separate room within 30 minutes.

### 2.4. Data Collection Tools

The data were collected using the three self-administered questionnaires: the demographic characteristics questionnaire, the Persian version of the International Physical Activity Questionnaire (IPAQ), and the Persian version of the Short-Form Health Survey Questionnaire (SF-36) [25, 26].

#### 2.4.1. The Demographic Characteristics Questionnaire

It consists of four questions about age, gender, education level, duration of T2DM, and medication treatment of T2DM.

#### 2.4.2. The Persian Version of the International Physical Activity Questionnaire (IPAQ)

This 21-item questionnaire was used to measure the level of physical activity. This instrument classifies the persons in three groups with low(not meeting medium or high criteria), medium (≥ 600 < 3000 MET-minutes per week), and high (≥ 3000 MET-minutes per week) physical activity. The ICC exceeded 0.7. In addition, the Spearman–Brown correlation coefficient was reported to be 0.9.

#### 2.4.3. The Persian Version of the Short-Form Health Survey Questionnaire (SF-36)

This tool was used to evaluate HRQOL. Cronbach's *α* coefficients ranged between 0.77 and 0.90 except for the vitality scale (*α* = 0.65). Convergent validity showed that all correlations above 0.40 ranged between 0.58 and 0.95. Factor analysis identified two principal components that jointly accounted for 65.9% of the variance. The original versions of both questionnaires were used for scoring [27, 28].

### 2.5. Data Processing and Analysis

After completing the questionnaires, the data were extracted and analyzed using the SPSS ver. 26 software [29]. Descriptive statistics, such as frequency, mean, and standard deviation, were used to describe the variables. In addition, Pearson's and Spearman's correlation, independent samples *T*-test, and one-way ANOVA were used to measure the correlation and relationship between the variables. Linear regression was performed and boxplot was drawn to explore the association between the variables. The significance level was set at 0.05.

## 3. Results

### 3.1. The Demographic Characteristics

The mean age of the 135 participants was 58.02 ± 9.4 years, and the mean duration of the disease was 8.81 ± 6.11 years. Furthermore, the majority of them were females and the minority of them had a university education and no medication treatment for diabetes (Table 1).

### 3.2. The Status of Physical Activity and HRQOL

The mean level of physical activity of participants was 1625.68 ± 1230.30 MET-min/week, and the majority of them were engaged in moderate physical activities (Table 1). Additionally, the mean level of HRQOL was 54 ± 27.63 (Table 2).

### 3.3. The Level of BMI and Metabolic Indicators

The mean level of BMI, FBS, HDL, LDL, triglyceride, total cholesterol, HbA1c, and systolic and diastolic blood pressure of participants was within the normal range (Table 2).

### 3.4. The Relationship between Variables

According to Table 3, Pearson's correlation test showed a significant linear relationship between physical activity (*r*: 0.20) and HDL level (*r*: −0.21) with HRQOL (*p* < 0.05). Moreover, a significant correlation was observed between gender and HRQOL (*r*: 0.27), education level, and physical activity (*r*: 0.34) (*p* < 0.01). In addition, LDL (*r*: −0.20), HbA1c (*r*: −0.21) level, and age (*r*: −0.21) correlated to physical activity (*p* < 0.05). But with the leveling of physical activity, the relationship between these factors and its levels was not seen. (Table 4).

### 3.5. The Association between HDL Level, Physical Activity, Gender, and HRQOL

**Figure 1:**
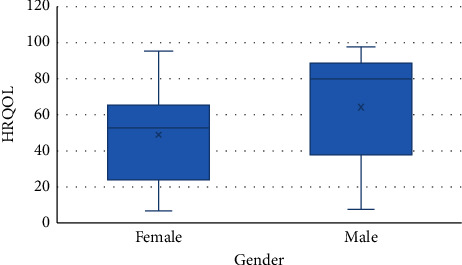
Association between gender and HRQOL.

**Figure 2:**
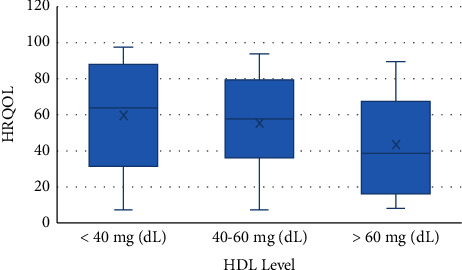
Association between HDL level and HRQOL.

**Figure 3:**
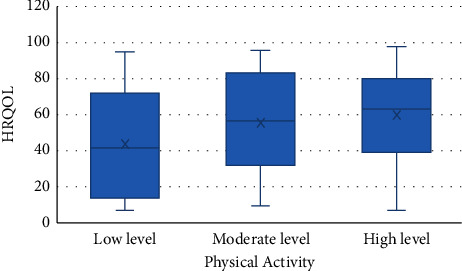
Association between physical activity and HRQOL.

**Figure 4:**
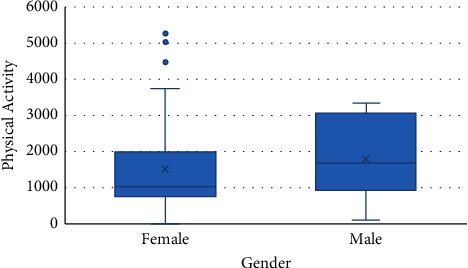
Relationship between gender and physical activity.

The results of multiple linear regression between gender, physical activity, and HDL level with HRQOL that were significant in Pearson's analysis are presented in Table 5. Accordingly, gender (*p* = 0.007) and HDL level (*p* = 0.02) were associated with QOL, so that its level was higher in males and lower in the group with HDL level >60 mg/dL (*p* = 0.03) (Figures 1 and 2). After adjusting physical activity for gender (*p* = 0.002), the association between it and HRQOL was demonstrated (Figure 3). According to Figure 4, men's physical activity was more intense than women's physical activity.

## 4. Discussion

Improving QOL is among the most important goal of the healthcare system; thus, it is important to investigate how diabetes affects patients' QOL [30, 31]. In the present study, a significant association was observed between HDL level >60 mg/dL and lower HRQOL, which was inconsistent with the results of Xepapadaki et al. [22], while some studies have shown that higher consumption of HDL is related to improved cardiovascular function, and lower consumption is associated with mental and cognitive disorders and subsequently better QOL. [32–34[Bibr B32][Bibr B33][Bibr B34]. In other studies, the high level of HDL was not confirmed as a protective factor for cardiovascular diseases, and the quality of HDL's function may be more important than its quantity for this matter [35, 36]. Hence, future research studies are needed to explore these relationships.

The present study showed no significant correlation between FBS, HbA1c, blood pressure, cholesterol level, and HRQOL, while other studies showed significant correlations using the 5-levelEuroQoL-5 dimensions (EQ-5D-5L) questionnaire or World Health Organization Quality of Life (WHOQOL-BREF) short-form questionnaire [21, 37, 38]. The difference in this correlation between the studies could be attributed to utilizing different questionnaires with different number of questions in the extent of dimensions on the relevant topic.

Another finding of our study was the association between male gender and higher HRQOL. Similarly, Castellano-Guerrero et al. and Huebschmann et al. had shown this relationship [39, 40]. It seems that the sociocultural and health behavior differences between men and women could be a reason for it.

In addition, the findings of the present study showed a significant association between the total score of HRQOL and the gender-adjusted physical activity, which was supported by Thiel et al.'s study [41]. Similarly, Xu et al. revealed a significant positive correlation between physical activity and QOL in patients with T2DM [42]. It should be noted that the best type of physical activity for people with T2DM is aerobic exercise, especially if it is done daily [43, 44]. This finding warns us to adopt appropriate supportive policies to improve education and provide environmental support for promoting physical activity in society, especially among women.

According to the findings of the present study, similar studies are recommended to be conducted on patients with chronic diseases in different age groups and population-based sampling methods. The results of this study can be effective in improving educational methods, as an effective factor in patients' self-management of chronic diseases.

The present study had some limitations including incomplete questionnaires, difficulties in having access to patients, and patients' low literacy levels that caused difficulties in completing the questionnaires. Other limitations were conducting the study in only one center, volunteer sampling, and data collection through self-report that could affect the generalizability of the results. In addition, the results might have been affected by personality differences and the lack of a healthy control group.

## 5. Conclusion

The results of the present study show that performing physical activity and regulating HDL levels could improve HRQOL in patients with T2DM. Therefore, according to the status of these factors in them, appropriate health programs such as self-directed group physical activity, team sports, and recreational sports, such as mountain climbing should be implemented to improve their QOL.

## Figures and Tables

**Table 1:** Demographic characteristics and physical activity level of the participants (*N* = 135).

Variable	Classification	Number (%)
Gender	Female	92 (68.1)
	Male	43 (31.9)

Age (years)	<45	11 (8.1)
	45–60	69 (51.1)
	≥60	55 (40.7)

Education level	Elementary-middle school degrees	63 (46.7)
	Higher than middle school degrees	61 (45.2)
	University degree	11 (8.2)

Duration of T2DM (years)	Below 5 years	48 (35.6)
	5–10 years	50 (37.0)
	More than 10 years	37 (27.4)

Medication treatment of T2DM	Yes	55 (40.4)
	No	80 (58.8)

Physical activity (MET-min/week)	Low physical activity	28 (20.7)
	Moderate physical activity	80 (59.3)
	High physical activity	37 (20.0)

T2DM: type 2 diabetes mellitus; MET: metabolic equivalent.

**Table 2:** Level of HRQOL and metabolic indicators of the participants (*N* = 135).

Variables	Mean ± standard deviation	Range
HRQOL	54 ± 27.63	7–97.78
BMI (kg/m^2^)	26.54 ± 3.68	20–41
HDL level (mg/dL)	54.59 ± 30.86	23–256
LDL level (mg/dL)	75.99 ± 29.18	26–186
FBS level (mg/dL)	153.28 ± 49.28	78–324
HbA1c level (mmol/mol)	7.64 ± 1.84	2.9–16
Total cholesterol level (mg/dL)	144.06 ± 34.05	43–237
Triglyceride level (mg/dL)	147.51 ± 67.51	31–455
Systolic blood pressure (mmHg)	121.60 ± 10.99	80–160
Diastolic blood pressure (mmHg)	78.30 ± 7.61	60–100

HRQOL: Health-Related Quality of Life; BMI: body mass index; HDL: high-density lipoprotein; LDL: low-density lipoprotein; FBS: fasting blood sugar.

**Table 3:** The relationship between metabolic indicators, demographic characteristics, physical activity, and HRQOL of participants.

Variables	Age	Gender^¢^	Education level^¢^	Duration of T2DM	Medication treatment of T2DM^¢^	HRQOL	BMI	HDL	LDL	FBS	HbA1c	Total cholesterol	Triglyceride	Systolic BP	Diastolic BP	Physical activity
Age	1	0.21^*∗*^	−0.32^*∗∗*^	0.23^*∗∗*^	−0.07	−0.01	−0.17^*∗*^	−0.03	0.10	−0.12	0.07	−0.03	0.11	0.09	−0.02	−0.21^*∗*^
Gender^¢^		1	0.18^*∗*^	0.02	−0.21^*∗*^	0.27^*∗∗*^	−0.11	−0.23^*∗∗*^	−0.04	−0.15	−0.17^*∗*^	−0.18^*∗*^	−0.11	0.16	0.14	0.16
Education level^¢^			1	−0.11	−0.06	0.14	0.05	−0.12	−0.15	0.06	−0.11	−0.00	−0.17^*∗*^	0.00	0.00	0.34^*∗∗*^
Duration of T2DM				1	0.09	−0.09	−0.02	−0.00	−0.20^*∗*^	0.15	0.23^*∗∗*^	−0.19^*∗*^	0.01	0.06	−0.03	−0.14
Medication treatment of T2DM^¢^					1	−0.04	−0.06	−0.10	−0.09	−0.00	−0.03	−0.07	−0.06	−0.08	−0.03	−0.00
HRQOL						1	−0.04	−0.21^*∗*^	−0.15	0.05	−0.01	−0.10	0.03	−0.09	−0.02	0.20^*∗*^
BMI							1	0.03	0.09	0.08	0.10	0.10	0.08	0.03	−0.01	−0.05
HDL								1	−0.00	0.12	0.06	0.25^*∗∗*^	0.11	0.03	0.04	−0.13
LDL									1	−0.02	0.17^*∗*^	0.42^*∗∗*^	0.09	0.02	0.09	−0.20^*∗*^
FBS										1	0.43^*∗∗*^	0.09	0.21^*∗*^	0.04	0.06	−0.09
HbA1c											1	0.08	0.07	0.01	0.14	−0.21^*∗*^
Total cholesterol												1	0.41^*∗∗*^	−0.06	0.02	−0.06
Triglyceride													1	0.07	0.05	−0.07
Systolic BP														1	0.61^*∗∗*^	−0.01
Diastolic BP															1	−0.04
Physical activity																1

T2DM: type 2 diabetes mellitus; HRQOL: Health-Related Quality of Life; BMI: body mass index; HDL: high-density lipoprotein; LDL: low-density lipoprotein; FBS: fasting blood sugar; BP: blood pressure. ^¢^Spearman's correlation analysis. ^*∗*^Correlation is significant at 0.05 level. ^*∗∗*^Correlation is significant at 0.01 level.

**Table 4:** The relationship between gender and physical activity with level of HRQOL and metabolic indicators (*n* = 135).

Gender						Physical activity			
Variables		Mean ± standard deviation	*t*	*P*value	Variables	Level	Mean ± standard deviation	*F*	*P*value
HRQOL	Female	49.01 ± 24.88	−2.94	<0.001	HRQOL	Low	43.52 ± 31.25	2.85	0.06
	Male	64.67 ± 30.39				Moderate	55.66 ± 25.79		
						High	59.93 ± 27.13		

HDL	Female	55.62 ± 29.82	0.56	0.57	LDL	Low	83.07 ± 38.26	2.26	0.10
	Male	52.40 ± 33.21				Moderate	76.66 ± 26.84		
						High	66.67 ± 23.23		

HbA1c	Female	7.80 ± 1.88	1.45	0.14	HbA1c	Low	8.06 ± 2.00	1.44	0.23
						Moderate	7.64 ± 1.90		
	Male	7.30 ± 1.74				High	7.21 ± 1.41		

Total cholesterol	Female	148.86 ± 32.96	2.43	0.06	Age	Low	61.46 ± 7.27	2.05	0.86
	Male	133.79 ± 32.45				Moderate	57.34 ± 9.98		
						High	56.48 ± 9.06		

HRQOL: Health-Related Quality of Life; HDL: high-density lipoprotein; LDL: low-density lipoprotein.

**Table 5:** Determinants of HRQOL.

Model	Estimate	Standard error	95% confidence interval	*P* value
Constant	39.05	8.43	22.37–55.73	<0.001
HDL level	−0.16	0.07	−0.31–−0.01	0.02
<40 mg/dL	Ref.		1.00	
40–60 mg/dL	−15.33	10.20	−35.52–4.85	0.13
>60 mg/dL	−20.31	9.54	−39.20–−1.42	0.03
Gender	13.21	4.82	3.67–22.75	0.007
Physical activity	0.003	0.002	0.000–0.007	0.08
Physical activity^*∗*^	0.007	0.002	0.002–0.01	0.002

HDL: high-density lipoprotein. ^*∗*^Adjusted for gender.

## Data Availability

The data used to support the findings of this study are available from the corresponding author upon request.
